# Childhood ultraviolet exposure and skin cancer prevention: A call for change in national policy in South Africa

**DOI:** 10.4102/jphia.v17i1.1895

**Published:** 2026-06-09

**Authors:** Magrietha Swart

**Affiliations:** 1 Department Anaesthetics, Independent Practice, Dr AP Swart Inc, Molteno Anaesthetics, Cape Town, South Africa

**Keywords:** skin cancer, ultraviolet exposure, prevention, public education, national health policy

## Abstract

Skin cancer incidence is rising both globally and in South Africa, and evidence shows that sunburns in childhood significantly affect the risk of developing melanoma in later life. Sun protection in childhood remains an underutilised avenue for preventive medicine in South Africa. In contrast to South Africa, countries like Australia have implemented numerous state and national sun programmes that have shown significant success, with declining melanoma rates in younger populations. South Africa observes annual skin cancer awareness campaigns but lacks a comprehensive, national school sun protection policy, and such policies are seldom implemented at school level. It is suggested that healthcare professionals engage in sun protection advocacy nationally. This includes patient education, targeted counselling for adolescents, engagement with communities and schools and the incorporation of routine skin examination into consultations and targeted screening of high-risk individuals. Reducing ultraviolet light exposure in the paediatric population offers a cost-effective and practical way to alleviate the burden of skin cancer in the coming decades.

## Introduction

The incidence of all skin cancers has increased globally over recent decades, with melanoma and non-melanoma skin cancers contributing substantially to the disease burden.^1^ Although the incidence of skin cancer is higher in patients of European descent, it affects all population groups. The reported incidence is 7.2–16.4 per 100 000, with melanoma being 2.9–4.9 per 100 000 population, although it is estimated that in general skin cancers in Southern Africa are underreported.^2^

South Africa’s skin cancer landscape is broader than ultraviolet (UV)-related melanoma alone. Human immunodeficiency virus (HIV)-associated malignancies, notably Kaposi sarcoma, contribute significantly to the country’s cancer burden; Africa carries the overwhelming majority of the global burden of HIV-attributable cancers.^3^ People with oculocutaneous albinism represent a particularly vulnerable group: studies from sub-Saharan Africa consistently show that individuals with albinism are at dramatically elevated risk of developing squamous cell carcinoma (SCC), often at a young age, and that sun protection in this population is critically inadequate.^2,4,5,6^ Non-melanoma skin cancers, particularly basal cell carcinoma (BCC) and SCC, rank among the most commonly diagnosed invasive cancers in South Africa, with melanoma also featuring in the top 10 diagnosed malignancies.^7^ Even if higher skin melanin content offers some sun protection, this should not lead to neglect of sun protection measures across all population groups.

Extensive epidemiological research into the pathophysiology of skin cancer has demonstrated that sunburns occurring in childhood carry higher risk for melanoma than sunburns acquired later in life.^8^ While skin cancer risk is cumulative across the life course, a substantial proportion of lifetime UV exposure occurs during childhood,^9^ making this a critical window for prevention. Framed this way, melanoma (and non-melanoma skin cancers) should not be regarded as an adult disease, but as a condition rooted in childhood exposure and preventable harm. Yet sun protection remains an underutilised and undervalued pillar of preventive medicine, particularly in South Africa. From a public health perspective, childhood sunburn represents a failure of prevention, one that demands coordinated national policy rather than reliance on individual responsibility alone.

## Australia as comparison for South Africa

Australia is used here as a comparison because of its successful sun protection programme in a comparably high-UV environment, and because it demonstrates what sustained, policy-driven prevention can achieve. While South Africa’s melanoma incidence currently shows no evidence of plateauing,^1^ a recent analysis of Australian melanoma incidence shows a plateau and even a decline in younger population groups (below age 40 years),^10^ widely attributed to their national skin cancer awareness campaign since the 1980s. Although the population demographics differ between the two countries, with patients of European descent being the largest group in Australia, the UV radiation exposure in the two countries is comparable and it can therefore illustrate differing policy outcomes and mechanism, though it does not however imply direct epidemiological equivalence.

South African studies have shown that most government schools lack formal sun protection policies, and no national SunSmart Schools accreditation programme currently exists,^11,12^ by marked contrast. In the wider sub-Saharan African context, targeted initiatives for people with albinism for whom UV protection is lifesaving have demonstrated that community-based prevention is feasible even in resource-constrained settings. In Tanzania, where albinism prevalence is among the highest in the world, community programmes have successfully produced affordable sunscreen locally and distributed protective clothing and education.^4,5^ Similar programmes have been documented in Malawi and Togo.^13,14^

South Africa, as a middle-income country, has the resources for a broader national policy programme, and the Australian experience shows what can be achieved. Even though South Africa has a very different demographic from Australia, with a high burden of HIV-related disease, oculocutaneous albinism inadequately receiving sun protection^6^ and a greater racial diversity, the benefits of a population-wide policy is clearly demonstrated in the Australian context. While outcomes will likely differ between South Africa and Australia based on these demographic factors, public health benefits can be expected for all South Africans, even if a one-to-one comparison to the Australian experience could not be made.

## Australian programmes

The Australian government has made sun protection part of their national health policy, with a higher incidence of skin cancer than South Africa,^15^ and their melanoma patients have a much better prognosis as education and policy leads to earlier detection.^2^ In the 1980s, Australia launched the Slip! Slop! Slap! Campaign funded by national health, and state education requires schools to have sun protection policies.^16^ Many schools have a *no hat, no play* policy and formally adopted UV-protective swimwear including swim shirts to protect against sun damage^17^ and scheduled outdoor activity outside peak UV periods. The Australian government also has regular national advertisement campaigns about sun protection, and made another 15 million dollars available in 2024 for this specific purpose.^18^ After the death of 26-year-old Clare Oliver in 2007 from melanoma skin cancer, the Australian government started to crack down on sunbed salons. In 2016 the Australian government banned all sunbeds in all provinces because of the clear and direct risk of developing sun cancer associated with regular use.^19^ As recently as January 2026, hundreds of medical professionals in Australia signed an open letter calling for ‘sunscreen application time’ during school hours, during which children would be allowed 5 min to apply their sunscreen.^20^

The results have been measured, and among Australians aged 15–24 years, age-standardised melanoma incidence fell from 92.2 per million in 1984–1988 to 41.5 per million in 2014–2018, representing a decline of more than 55%. For those aged 30–39 years, incidence dropped from 31.4 per 100 000 in 2000 to 23.6 per 100 000 in 2024.^21^ Melanoma mortality in the 20–44 year age group has declined by approximately 42% – 48% over the same period.^22^ These declines in younger cohorts are widely attributed to sustained sun protection campaigns targeting children and adolescents, demonstrating that behavioural change driven by consistent policy and education on a national level can bring meaningful improvements to the disease burden.

## South Africa

South Africa observes an annual SunSmart Skin Cancer Awareness Month as part of the National Department of Health’s official awareness calendar.^23^ This, however, falls short of a sustained, comprehensive and nationally implemented school sun protection policy or accreditation programme and does not constitute a comprehensive prevention strategy. The Cancer Association of South Africa (CANSA) has advocated for such programmes, but they are a non-governmental organisation (NGO) and do not influence national policy. Despite repeated calls from CANSA for regulation and/or banning of the use of sunbeds,^24^ this advice has gone unheeded.

Government-run schools do not have any legislated policies that directly protect children from excessive sun exposure. Some surveys were conducted in government-run schools illustrating attitudes to sunscreen and sun exposure, as well as knowledge and practice among schoolchildren, which showed inadequate knowledge and frequent reported sunburn.^11,12^ In South Africa, most schools start school sport activities in the afternoons during peak UV hours, when any sunscreen that a prudent parent applied that morning, is now inactive. The use of hats is not widespread, and many schools do not include hats in their sports uniform. Protective clothing, including UV-protective swimwear, represents a simple and practical intervention to reduce UV exposure during outdoor and aquatic activities. It has to be acknowledged that there is a difference in melanoma distribution by population group, such as acral melanoma affecting the palms, soles and nailbeds being the predominant subtype among black South Africans, and sun protection recommendations should be framed with this in mind.^25^

The affordability of sun protection is also a socioeconomic factor that needs to be considered. In South Africa, sunscreen is priced as a cosmetic product rather than a preventive health measure, placing it beyond the financial reach of many families. This creates an inequity in cancer prevention that national policy could directly address.

South Africa has demonstrated that it can implement cancer-preventive strategies in ways that are both feasible and effective. The national cervical cancer prevention strategy, that includes age-based screening, health education and HPV-vaccinations of school children, demonstrates that structured preventive approaches can be effective if the political will exists.^26^ South Africa’s anti-tobacco legislation, which explicitly targets adolescents through school-based restrictions and public awareness campaigns, provides a further domestic precedent for using policy to protect young people from a preventable cancer risk. Skin cancer prevention campaigns do not require any novel infrastructure, but rather, national education programmes, school policies and regulation. Baseline surveys for assessing efficacy have been shown feasible.^12^ It does not require expensive technological advances or even extra research beyond assessing the efficacy of the implementation of the campaign, because the evidence is clear: childhood UV radiation exposure is a major risk in the development of skin cancer. This suggests a gap in prioritisation rather than capacity.

### Recommendations

What can we do as healthcare professionals?

**• Advocacy at a national level**

We can advocate through established organisations such as CANSA to support the development of coordinated, national sun-protective strategies and the banning of the use of sunbeds. Collective advocacy through professional and cancer organisations carries far greater influence than individual efforts. The National Department of Health and the Department of Basic Education should be called on to develop and implement a formal national school sun protection policy (Box 1)^27,28^. Advocating for sunscreen prices to be reduced through zero value-added tax (VAT), bulk purchasing for schools or inclusion into a national preventive programme would address affordability and lessen the inequality in cancer prevention. Increasing the availability and reducing the cost of UV-protective clothing is also important.

BOX 1 Recommended school-level sun protection interventions.

- Shade structures in playgrounds and sports areas.
- Rescheduling of outdoor activities to avoid peak UV exposure (10:00–14:00).
- Mandatory hats as part of school uniform and sports uniform (‘no hat, no play’).
- Provision of broad-spectrum sunscreen (SPF ≥ 30) in classrooms, accessible before afternoon sport.
- Promotion of UV-protective clothing and swimwear for all learners.
- Staff role-modelling of sun-safe behaviour.
- Integration of sun-safety education into the school curriculum.
  *Source*: Adapted from Tod B, Whitaker D, Visser W, et al. Integrated sun protection advice for the South African population. Int J Dermatol. 2024;63(3):277–287. https://doi.org/10.1111/ijd.16980; World Health Organization. Sun protection: An essential element of a health-promoting school. Geneva: WHO; 2003.
  SPF, Sun Protection Factor; UV, ultraviolet.

**• Routine patient and parent education**

Ultraviolet protection should form part of routine preventive counselling, particularly during paediatric consultations. Parents and children should be advised to use a broad-spectrum sunscreen of at least Sun Protection Factor (SPF) 30, applied before school and reapplied every 2 h – 4 h during outdoor activities and sport. The routine use of hats and UV-protective swimwear should be actively encouraged.

**• Targeted counselling for adolescents**

With teenagers, discussions around sun protection should include the effects of photo-ageing, as this is often a more immediate and persuasive concern. Misconceptions around sunscreen and acne should be addressed, with reassurance that non-comedogenic formulations are widely available.

**• Creating a sun-aware practice environment**

Displaying clear, evidence-based educational posters in consulting rooms and waiting areas helps normalise sun protection as a medical priority and reinforces verbal counselling. Recent integrated sun protection guidance for the South African population provides a locally relevant resource for clinical environments.^27^

**• Community and school engagement**

Healthcare workers can extend their influence beyond the consultation room by engaging with local schools, offering talks or guidance on the importance of sun-protective policies during sport, outdoor activities and swimming.^28^ Schools can be encouraged to have a large sunscreen bottle in each class which children could access prior to afternoon sport. Education can also be extended to placing posters at community pools and sport arenas.

**• Early detection through targeted skin examination**

Opportunistic skin examinations should be incorporated into routine care for high-risk individuals, including those with fair skin, oculocutaneous albinism, immunosuppression such as HIV, a history of significant sun exposure or previous non-melanoma skin cancer. Early detection remains a critical component of improving outcomes.

## Conclusion

Skin cancer is a global problem with an increasing incidence worldwide, including in South Africa. It is not an inevitable disease dependent on your geographical location and skin type, but rather, with proper education, intervention and policy, it is a largely preventable disease. South Africa’s lack of national policy is in stark contrast to international communities, such as Australia or other African contexts, that have demonstrated that early sun protection can change disease trajectories, making it a neglected area of preventive medicine, particularly in the paediatric population. South Africa already has an effective cervical cancer programme that proves it can implement large-scale preventive policies in children. As clinicians, we are in a unique position to work alongside advocacy groups, as well as advocate in our workplace and in our communities.

If childhood sun exposure continues to be neglected, South Africa will face an escalating and entirely preventable burden of skin cancer in the decades to come. The evidence is clear, the tools are available and the opportunity for intervention is now.
